# Genome-wide genotype-serum proteome mapping provides insights into the cross-ancestry differences in cardiometabolic disease susceptibility

**DOI:** 10.1038/s41467-023-36491-3

**Published:** 2023-02-16

**Authors:** Fengzhe Xu, Evan Yi-Wen Yu, Xue Cai, Liang Yue, Li-peng Jing, Xinxiu Liang, Yuanqing Fu, Zelei Miao, Min Yang, Menglei Shuai, Wanglong Gou, Congmei Xiao, Zhangzhi Xue, Yuting Xie, Sainan Li, Sha Lu, Meiqi Shi, Xuhong Wang, Wensheng Hu, Claudia Langenberg, Jian Yang, Yu-ming Chen, Tiannan Guo, Ju-Sheng Zheng

**Affiliations:** 1 School of Life Sciences, Fudan University, Shanghai, China; 2 School of Life Sciences, Westlake University, 310024 Hangzhou, China; 3 Key Laboratory of Environmental Medicine and Engineering of Ministry of Education, Department of Epidemiology & Biostatistics, School of Public Health, Southeast University, 210009 Nanjing, China; 4 Westlake Intelligent Biomarker Discovery (iMarker) Lab, Westlake Laboratory of Life Sciences and Biomedicine, 310024 Hangzhou, China; 5 Guangdong Provincial Key Laboratory of Food, Nutrition and Health, Department of Epidemiology, School of Public Health, Sun Yat-sen University, 510275 Guangzhou, China; 6 Institute of Epidemiology and Statistics, School of Public Health, Lanzhou University, 73000 Lanzhou, China; 7 Hangzhou Women’s Hospital (Hangzhou Maternity and Child Health Care Hospital), Hangzhou, China; 8 MRC Epidemiology Unit, Institute of Metabolic Science, University of Cambridge School of Clinical Medicine, Cambridge, CB2 0QQ UK; 9 Computational Medicine, Berlin Institute of Health at Charité–Universitätsmedizin Berlin, Berlin, 10117 Germany; 10 Westlake Laboratory of Life Sciences and Biomedicine, 310024 Hangzhou, China; 11 Institute of Basic Medical Sciences, Westlake Institute for Advanced Study, 310024 Hangzhou, China; 12 Research Center for Industries of the Future and Key Laboratory of Growth Regulation and Translational Research of Zhejiang Province, School of Life Sciences, Westlake University, 310030 Hangzhou, China

**Keywords:** Genome-wide association studies, Proteomics

## Abstract

Identification of protein quantitative trait loci (pQTL) helps understand the underlying mechanisms of diseases and discover promising targets for pharmacological intervention. For most important class of drug targets, genetic evidence needs to be generalizable to diverse populations. Given that the majority of the previous studies were conducted in European ancestry populations, little is known about the protein-associated genetic variants in East Asians. Based on data-independent acquisition mass spectrometry technique, we conduct genome-wide association analyses for 304 unique proteins in 2,958 Han Chinese participants. We identify 195 genetic variant-protein associations. Colocalization and Mendelian randomization analyses highlight 60 gene-protein-phenotype associations, 45 of which (75%) have not been prioritized in Europeans previously. Further cross-ancestry analyses uncover key proteins that contributed to the differences in the obesity-induced diabetes and coronary artery disease susceptibility. These findings provide novel druggable proteins as well as a unique resource for the trans-ancestry evaluation of protein-targeted drug discovery.

## Abstract (web summary)

Integrating genetic information with circulating proteomics can help understand mechanisms of disease. Here, the authors conduct genome-wide association analyses of the serum proteome in 2,958 Han Chinese individuals, uncovering proteins which may contribute to ancestry differences in cardiometabolic disease susceptibility.

## Introduction

Circulating proteins, as representatives of intermediate molecular phenotypes in human health, are widely used to reveal novel drug targets and translational biomarkers for clinical outcomes^1^. Genetic modulation on proteins has been well-known but poorly described, which has stimulated not only commercial but also scientific interest in integrating genetics and proteomics for providing new insights into human health.

In recent years, studies of blood-based protein quantitative trait loci (pQTL) using aptamer-based multiplex protein assay (SOMAscan) and antibody-based multiplex immunoassays (Olink panels) have identified thousands of associations between single-nucleotide polymorphisms (SNP) and protein levels, many of which colocalized with association signals for common human diseases^2–12^. Both techniques rely on conserved binding regions of protein epitopes, which possibly introduce binding artifacts. Therefore, cross-platform, e.g., using mass spectrometry (MS)-based technique, is desirable but still mainly lacking. In addition, the majority of investigations of pQTLs to date have been undertaken in populations of European ancestry, with few studies in non-Europeans, such as Africans or East Asians^10,13^.

In this study, we provided insights into the genetic control on circulating proteome in Han Chinese, by using data-independent acquisition (DIA) mass spectrometry, a high-throughput proteomics strategy that could accurately quantify proteins with high reproducibility in a complex proteome^14,15^. Furthermore, we performed a colocalization analysis of *cis*-pQTLs and complex traits/diseases followed by Mendelian randomization analysis, through which we observed the putative effect of proteins on clinically relevant phenotypes, suggesting causal roles and potential therapeutic targets of several proteins on certain diseases. Lastly, we demonstrated that our datasets could potentially help interpret the differences in diseases susceptibility between East Asians and Europeans, revealing a striking different obesity-induced proteomics signatures between the two populations with different ancestries. These results provided mechanistic insights into the different susceptibilities in obesity-induced diabetes and coronary artery disease risk in the East Asians compared with the Europeans.

## Results

### Associations of the genetic variants with proteins

**Fig. 1:**
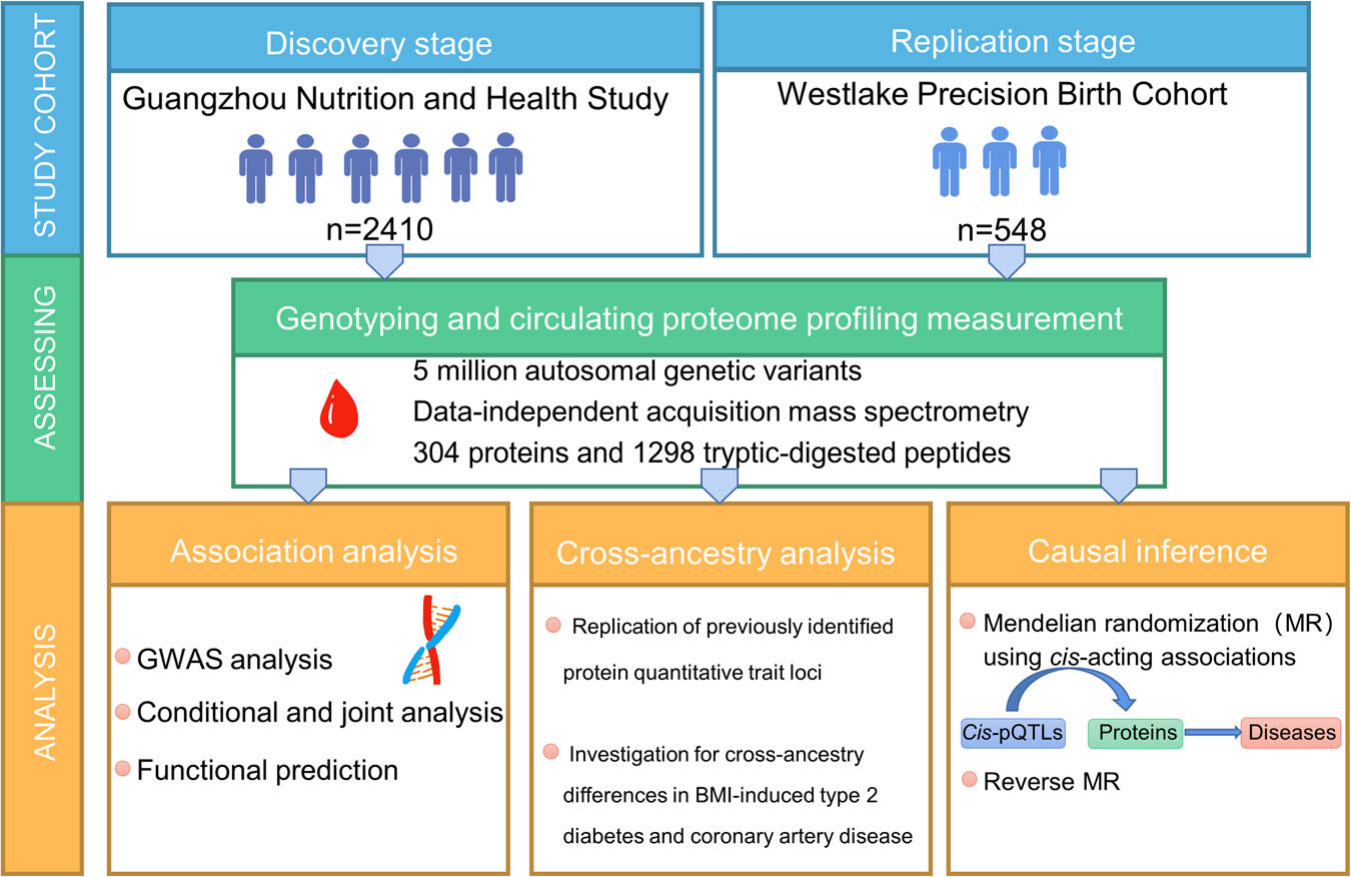
Overview of the study design. Using data-independent acquisition mass spectrometry, we measured serum proteome in up to 2410 Han Chinese participants with replication in 548 Han Chinese women. A total of 1298 tryptic-digested peptides and 304 proteins were included in the analysis. We used the colocalization of *cis*-pQTLs with the clinically relevant phenotypes, as well as the Mendelian randomization approach, to investigate the putative effects of the circulating proteins on complex traits/diseases. pQTL protein quantitative trait loci.

**Fig. 2:**
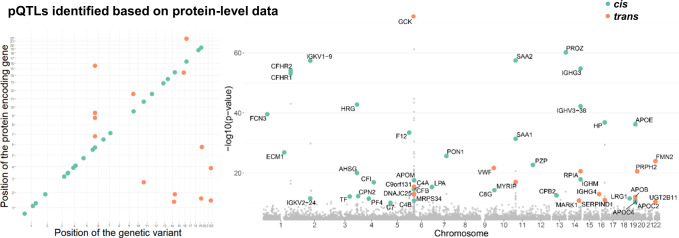
Gene–protein associations based on protein-level data. The left plots show the position of genetic variants against the position of the coding gene. The Manhattan plots (right) show the sentinel pQTLs and associated proteins. The green dots represent *cis*-pQTLs, while the red dots represent *trans*-pQTLs. pQTLs, protein quantitative trait loci. The genome-wide significant associations that should have (i) meta-analysis *P* < 5 × 10^−8^/304; (ii) *P* < 0.05 in four sub-cohorts; (iii) consistent direction of effect across the sub-cohorts. pQTLs protein quantitative trait loci.

We employed a mixed linear model-based genome-wide association analysis (GCTA-MLMA) of approximately 5 million autosomal variants against levels of 304 proteins in four sub-cohorts of GNHS independently^16,17^, which were then pooled by a meta-analysis with a random-effects model consisting of 2410 Chinese participants (Fig. 1, see below, Supplementary Data 1 and Supplementary Data 2). The missing data were imputed by 1/2 of the minimum measured value for the protein matrix. We identified genome-wide signals for 48 proteins, and extracted the most significant lead SNP of each protein as its pQTL (*P* < 1.6 × 10^−10^, 5.0 × 10^−8^/304, “Methods”) (Fig. 2 and Supplementary Data 3). We defined the pQTLs located within 1 Mb distance to the transcript starting sites (TSS) of the corresponding genes as *cis*-acting variants, while the ones out of 1 Mb to TSS as *trans*-acting variants. Overall, 34 are *cis*-pQTLs (71%), and 14 are *trans*-pQTLs (29%). To detect secondary signals at the same locus, we conducted a stepwise conditional analysis by GCTA-COJO^18^, using the same threshold of the genome-wide significance, and observed seven additional pQTLs for four proteins (“Methods” and Supplementary Data 5).

**Fig. 3:**
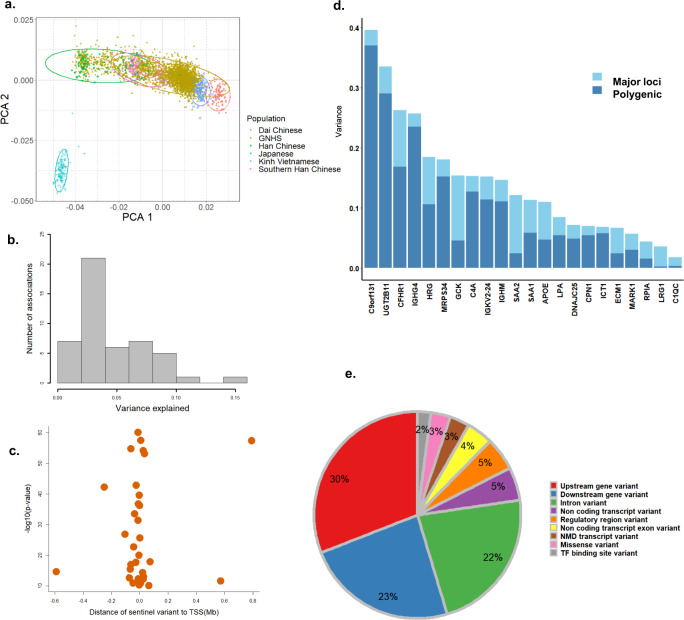
Characteristics of sentinel pQTLs. **a** Genetic principal component of the GNHS study compared to the 505 East Asian participants from the 1000 Genomes Project Phase3. **b** Distribution of explained variance that the genetic variant contributed to the corresponding protein. **c** The distance of lead variant to the transcript start site. **d** Heritability of circulating proteins. The variance explained by the lead SNPs is shown in light blue, with the variance explained by the polygenic background shown in dark blue. **e** The proportion of predicted functional annotation classes of the identified genetic variants. GNHS Guangzhou Nutrition and Health Study, PCA principal component analysis, TSS transcript start site, pQTLs protein quantitative trait loci.

The median value of variance explained by an independent lead genetic variant was 0.034 (ranging from 0.01 to 0.14), 14 of 48 lead genetic variants were shown to explain more than 0.05 of variance (Fig. 3b). We found that the 48 lead SNPs and the 7 additional independent SNPs (identified from the conditional analysis), including both *cis-* and *trans-*pQTLs, could explain 11–15% of the variance of corresponding proteins, of which 2.6–7.8% were contributed by the seven additional SNPs (Supplementary Data 5). In total 91.6% of the *cis*-pQTLs were located in regions within 0.2 Mb to TSS (Fig. 3c). Furthermore, we estimated the phenotypic variance contributed by the lead pQTLs, and the heritability explained by the additional genome-wide SNPs located over 10 Mb to the lead pQTLs (i.e., the polygenic background) (“Methods”). We found that for some proteins, the polygenic background explained a higher level of heritability than the lead pQTLs, whereas for some proteins such as hexokinase-4 (GCK) and serum amyloid A-2 protein (SAA2), the major loci contributed more than the polygenic background (Fig. 3d and Supplementary Data 4). Among all the identified pQTLs, intronic variants accounted for 22%, variants located at the 5’-region of a gene (upstream genetic variants) accounted for 30%, and variants located at the 3’-region of a gene (downstream genetic variants) accounted for 23% (Fig. 3e).

### Complementary GWAS analysis based on peptide-level data

Next, we employed tryptic-digested peptide-level data to perform the complementary GWAS analyses in addition to the above GWAS based on protein-level data. We included 1298 peptides that were only mapped to one protein, with an average of 4.3 peptides (ranging from 1 to 89 peptides) per protein. Based on peptide-level profiling, we found 147 pQTLs for 64 proteins at *P* < 3.9 × 10^−11^ (5.0 × 10^−8^/1298, “Methods”). Of 64 proteins, the pQTLs of 19 proteins have not been identified using protein-level data. Except for apolipoprotein B (APOB), the rest of the peptides were found to be genetically associated with SNPs within the same locus to their corresponding proteins. For APOB, one peptide was associated with a *cis*-acting locus, while nine peptides shared the *trans*-acting locus. In addition, 122 (83%) out of 147 pQTLs could be reproduced using protein-level data at false discovery rate (FDR) <0.05. Furthermore, no correlation (rho = 0.05) was observed between the number of peptides with the genetic association and the reproducibility (i.e., the gene–protein associations could be successfully replicated using protein-level data).

### Sensitivity analysis and replication of the identified pQTLs

**Fig. 4:**
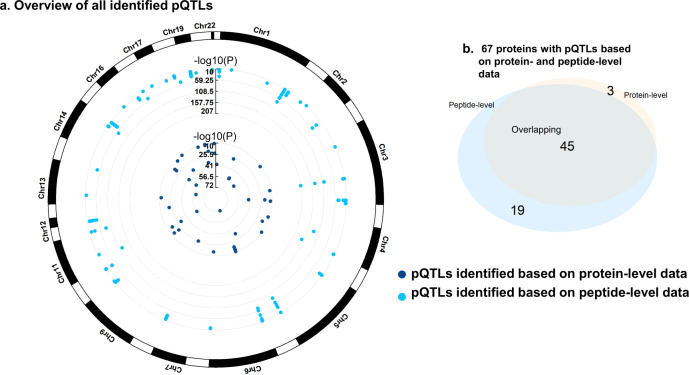
Genomic atlas of all identified pQTLs. **a** Overview of all identified proteins excluding the participants with missing data in each protein or peptide. Each dot represents a protein/peptide-associated genetic variant. The genome-wide significant associations that should have (i) meta-analysis *P* < 5 × 10^−8^/*n*, where *n* is the number of proteins/peptides; (ii) *P* < 0.05 in four sub-cohorts; (iii) consistent direction of effect across the sub-cohorts. **b** Number of proteins identified by protein- or peptide-level data. We found 67 proteins with pQTLs in Han Chinese, three of which were based on protein-level data and 19 on peptide-level data. pQTLs protein quantitative trait loci.

We performed a sensitivity analysis for 195 pQTLs (48 protein-level pQTLs and 147 peptide-level pQTLs, Fig. 4) by excluding the participants with missing data in each protein or peptide and found that the results were largely similar to the main model with imputation, whereof only 11 pQTLs failed to reach significance at FDR < 0.05 (8 *cis*-acting variants, 3 *trans*-acting variants) (Supplementary Data 3). Also, we calculated the statistical power for each association in 195 pQTLs, whereof 146 (75%) associations were justified with sufficient statistical power (>0.8) (Supplementary Data 3). To examine whether the pQTLs could be replicated, we measured the serum proteome in an independent cohort study consisting of 548 Chinese women. Among all identified pQTLs, 38 out of 39 (97%) *trans*-pQTLs and 153 out of 156 (98%) *cis*-pQTLs could be replicated with sufficient statistical power (>0.8). Despite the replication cohort being made up of women who were younger than the participants in the discovery cohort (mean age: 31.1 versus 63.4 years), we replicated 165 (84.6%) out of 195 pQTLs at FDR < 0.05 (Supplementary Data 6). These observations suggested that GWAS analyses using both MS-based protein-level and peptide-level data were effective and appropriate for identifying possible pQTLs.

### *Trans*-ancestry and cross-platform replication of previously reported pQTLs

To test whether the prior reported pQTLs among European population could be replicated in our Chinese population, we firstly checked the overlapped proteins and found that among the 304 proteins measured in the present Chinese study, 132 proteins had reported pQTLs among Europeans based on SOMAscan assay (130 proteins)^8,12,19,20^, Olink assay (1 protein)^5^ or MS (3 proteins, 2 of them accessible through SOMAscan assay)^21^. Of the 132 proteins and related pQTLs (i.e., independent lead SNPs) identified previously among Europeans, there were 1249 pQTLs from 118 proteins available in our present Chinese population for replication. The 1249 pQTLs showed moderately correlated effect sizes between prior European studies and our current Chinese study (*r* = 0.41). Of them (i.e., 1249 pQTLs), 197 (16%) associations were replicated in our Chinese study with consistent directions of effect (FDR < 0.05, Supplementary Data 7), including 135 *cis*-pQTLs and 62 *trans*-pQTLs.

Then, we tried to replicate our newly identified 195 pQTLs in the prior European pQTL datasets, in which 111 pQTLs were available^20^. We found that 62% (69/111) associations could be validated in the European populations at FDR <0.05^20^, suggesting that many pQTLs identified in the current study may be conservative across the two populations (Supplementary Data 6).

Three pQTLs, corresponding to haptoglobin (HP), alpha-1-antitrypsin (SERPINA1) and apolipoprotein E (APOE), were previously identified by an MS-based proteomics study^21^ consisting of 1060 European-descent individuals, showing consistent directions of effect with significance at FDR <0.05 in our study. In addition, we found that for some proteins (e.g., APOE, AHSG, and HP), the directions of effect were opposite in our discovery cohort compared to the previously affinity-based studies^12,19^. For instance, T-allele of rs7412 was positively associated with APOE levels in our study, consistent with the results from a previous meta-analysis reporting that the increased number of T-allele mutation of rs7412 was associated with a higher level of APOE^22^, while an inverse association was observed in an aptamer-based pQTL European study^12^.

### Integrative analysis of pQTLs with clinically relevant phenotypes

**Fig. 5:**
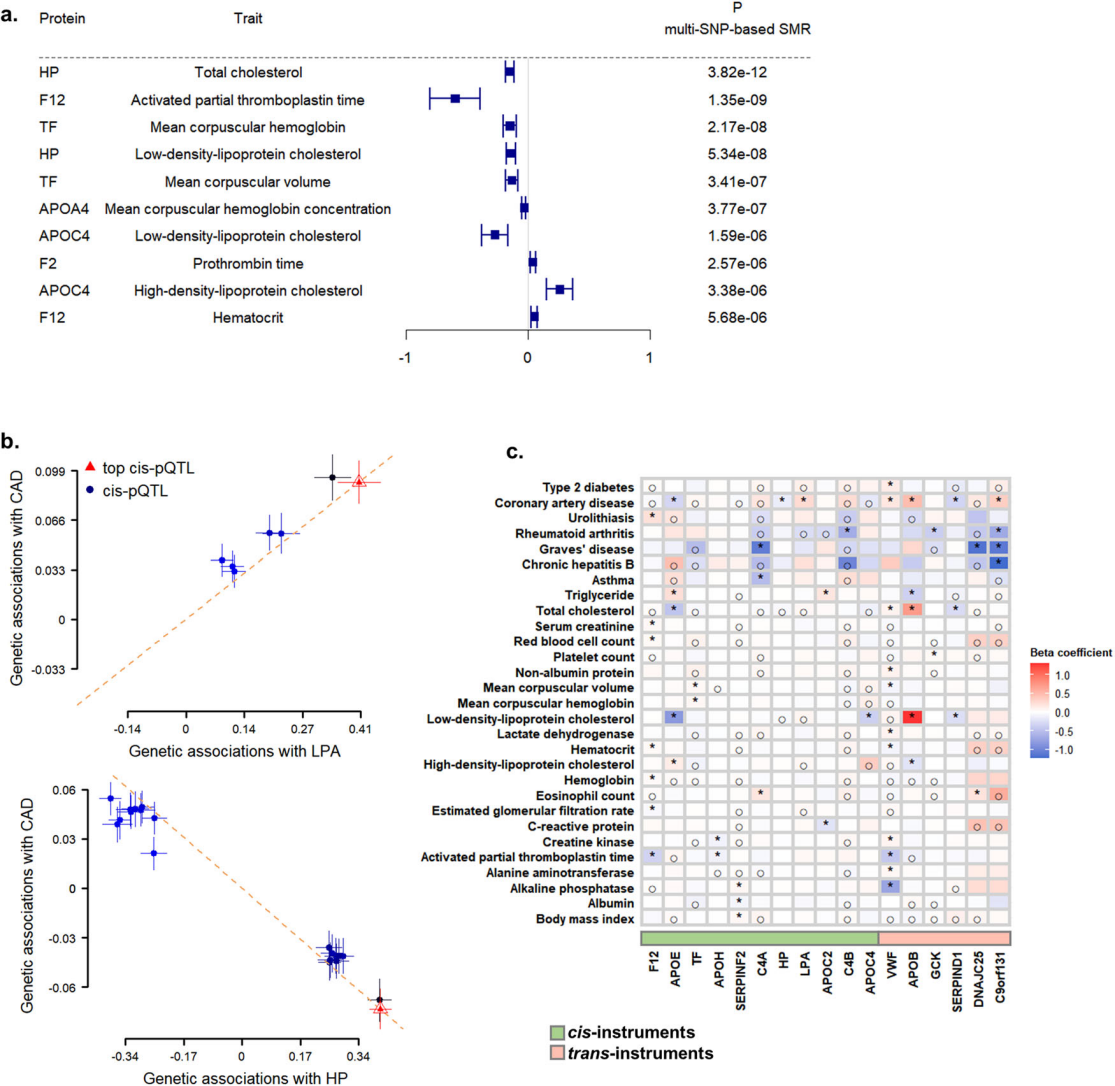
Associations between proteins and clinically relevant phenotypes. **a** Colocalization of *cis*-pQTLs and the clinical traits. The squares represent the estimated effect size from the summary-data-based Mendelian randomization analysis, and the lines represent the 95% confidence intervals. **b** Effect sizes from disease GWAS studies against those from pQTL summary statistics. The orange dashed lines show the estimate at the top *cis*-pQTL. The error bars represent the standard errors of SNP effects. **c** Putative causal relationships between serum proteins and clinically relevant phenotypes. The clinical traits were obtained from GWAS summary statistics of BioBank Japan. The green represents proteins with *cis-*instruments, while the red represents proteins with *trans-*instruments. ^○^*P*_raw_ < 0.05; *P_Bonferroni_ < 0.05. GWAS genome-wide association analysis, SMR summary-data-based Mendelian Randomization, CAD coronary artery disease.

We used the multi-SNPs-based SMR (summary-data-based Mendelian randomization) test and HEIDI (heterogeneity in dependent instruments) analysis^23,24^ to assess the causal inference of *cis*-QTLs with clinically relevant phenotypes. We extracted *cis*-pQTLs identified at *P* < 5 × 10^−8^ and obtained the GWAS summary statistics of outcomes from external datasets, with 57 clinical traits and 35 diseases^25–27^ from the Biobank Japan (BBJ) study, and the summary statistics of type 2 diabetes from the AGEN-T2D study^28^. Despite we observed the presence of population stratification between Chinese and Japanese (Fig. 3a), Han Chinese and Japanese populations are usually considered together as East Asians, thereby with the rationale to be referered to each other for MR analysis. After excluding the pQTLs located at the major histocompatibility complex (MHC) region, we retained 31 proteins and 160 peptides with correspondence to 51 proteins. We found 43 associations comprising 7 proteins and 10 traits that passed the HEIDI test (*P*_HEIDI_ < 0.05) and experiment-wise significance threshold corrected for the multiplication of 51 proteins and 93 traits (*P*_SMR_ < 1.1 × 10^−5^, i.e., 0.05/4743) (Fig. 5a and Supplementary Data 8). The results showed that increased levels of apolipoprotein (a) (LPA) were significantly associated with a higher risk of coronary artery disease (CAD) (odds ratio = 1.26, *P* = 2.1 × 10^−7^) (Fig. 5b). In addition, the levels of HP were negatively associated with CAD risk at the borderline experiment-wise significance (odds ratio = 0.84, *P* = 1.4 × 10^−5^) (Fig. 5b). The HP was also inversely associated with CAD-related traits, e.g., LDL-c and total cholesterol, in which the association between the HP and LDL-c was consistent in the European populations (Beta = −0.058, *P* = 5.1 × 10^−7^)^29^. Furthermore, APOE, which is essential in the development of cardiovascular and neurodegenerative diseases, had a nominally positive association (*P* < 0.05) with esophageal cancer and hematological malignancy (Supplementary Data 8). The carboxypeptidase B2 (CPB2) showed a nominally negative association (*P* < 0.05) with the risk of neurological disorders including cerebral aneurysm and epilepsy.

### Mendelian randomization analysis for putative causal relationships

To clarify the potential causal effects of protein levels on diseases, we performed two-sample Mendelian randomization analysis by integrating *cis-*pQTLs clumped at *P* < 5 × 10^−8^ (LD *r*^2^ < 0.05) and lead *trans*-acting variants as instrumental variables (Methods and Supplementary Data 10). We used the Generalized Summary-data-based Mendelian Randomization (GSMR) and HEIDI methods to perform the forward and reverse MR analysis^30^, and the outcome variables were derived from the BBJ study and the AGEN-T2D study^25–28^. The advantage of GSMR and HEIDI test is their high statistical power in detecting pleiotropic effects. This analysis included 41 proteins and 179 tryptic-digested peptides, corresponding to 64 proteins, 16 of which have yet to be studied before^5,8,12,19,20^.

We found that the genetically determined von Willebrand factor (VWF) levels may increase the risk of CAD and type 2 diabetes (T2D) (*P* < 8.4 × 10^−6^, i.e., 0.05/5952 computed by 64 proteins and 93 traits, Fig. 5c and Supplementary Data 9). The *trans-*pQTLs associated with VWF levels were located in the *ABO* gene, and previous studies suggested that ABO blood groups were associated with several health and disease outcomes^31,32^, e.g., hyperlipidemia, T2D, and heart failure. The top *trans*-pQTL (rs687621) for VWF was in LD with genetic variants that determined blood types (rs8176719, *r*^2^ = 0.95; rs8176746, *r*^2^ = 0.34), which provided the genetic underpinning and possible mechanism underlying the links between ABO blood groups and cardiometabolic health. According to transcriptomics data from the Human Protein Atlas Version 20.1 (see refs. ^33,34^), *PRPH2*, mainly expressed in the retina, showed a positive association with glaucoma risk (odds ratio = 1.11, *P* = 1.8 × 10^−2^), which indicated that pQTL in plasma may contain the information on the role of proteins expressed in specific tissues in the development of disease.

Furthermore, the results of MR analysis showed that some proteins were associated with metabolic traits, for instance, IGHG4 was inversely associated with left ventricular mass and left ventricular mass index that had been used to predict abnormal cardiovascular events^35^ (Supplementary Data 9). Through reverse MR analysis, we found a positive association of rheumatic arthritis with C4A and C4B. In addition, metabolic traits, e.g., LDL-c, lactate dehydrogenase (LDH), and albumin/globulin ratio (AG), were associated with specific proteins (Supplementary Data 11).

### Druggable targets pinpointed by proteins for complex traits

**Fig. 6:**
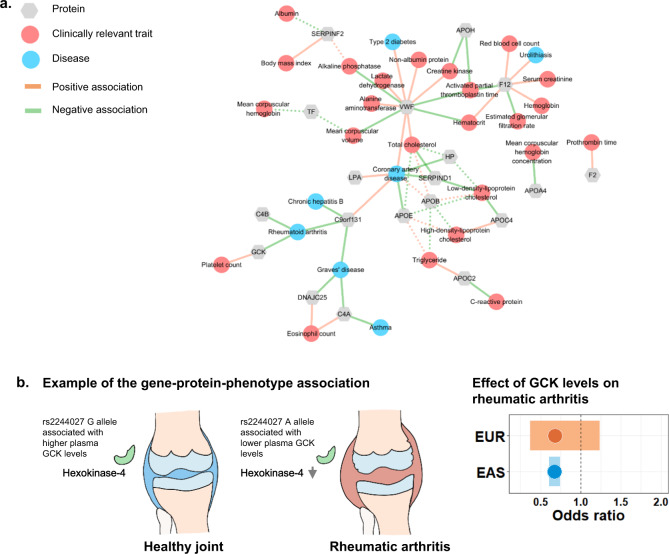
Network representation of potential gene–protein–phenotype associations. **a** Associations between proteins and diseases, as well as clinically relevant traits found by Mendelian randomization analysis and colocalization analysis (*P* < 0.05 after Bonferroni correction). The solid line represents the gene–phenotype connections that have yet to be prioritized in Europeans, whereas the dashed lines represent those that have already been reported. The color of the line denotes the effect directions (orange, positive associations; green, negative associations). Proteins are represented by the gray dots, whereas diseases and traits are represented by the blue and red dots, respectively. **b** An example from the gene–protein–phenotype map. Higher hexokinase-4 (GCK) levels are associated with a lower rheumatic arthritis risk. The plot shows the consistent effect of GCK on rheumatic arthritis across two populations. The effect sizes are present as the odds ratio per higher RINT(GCK). EAS East Asian, EUR European, RINT rank-based inverse normal transformation.

Based on the above colocalization and MR analyses, we identified 19 putative druggable proteins (*P* < 0.05 after Bonferroni correction) for 7 diseases and 24 clinically relevant traits, with a total of 60 protein–phenotype associations (Fig. 6a and Supplementary Data 12). In total, 45 (75%) out of the 60 associations were novel and have not been prioritized in Europeans^8^. For instance, the genetic variants at the MHC region could regulate the expression levels of hexokinase-4 (GCK), and the genetically determined higher GCK levels were associated with a lower risk of rheumatoid arthritis in East Asians (Fig. 6b) (odds ratio = 0.67, *P* = 1.2 × 10^−13^). According to a published animal study^36^, hexokinase is a pattern-recognition receptor for innate immunity. We could replicate the effect of GCK on rheumatoid arthritis in Europeans (Fig. 6b, odds ratio = 0.68, *P* = 0.21) based on the published GWAS results^20,37^. Our results suggested the possible relationships between hexokinase-4 and autoimmune diseases in humans.

In addition, coagulation factor XII (F12) was a newly identified therapeutic target for kidney diseases. F12 has been a candidate target for thromboembolic and inflammatory diseases^38^. In our current study, we observed the associations of F12 with estimated glomerular filtration rate (EGFR), serum creatinine, and urolithiasis. Furthermore, we summarized the protein–phenotype associations with supportive evidence of both colocalization and Mendelian randomization analyses (Supplementary Data 15). Of them, alpha-2-antiplasmin (SERPINF2) was positively associated with BMI and C-reactive protein, suggesting that SERPINF2 was a potential therapeutic target in metabolic diseases.

Most of the proteins that share genetic underpinnings with those tested diseases are currently unavailable as targeted drugs based on the DrugBank database (v5.1.8)^39^, while their expression or activation could be modulated by several common small molecules such as zinc and copper (Supplementary Data 13).

### Interpretation for the *trans*-ancestry cardiometabolic disease susceptibility

**Fig. 7:**
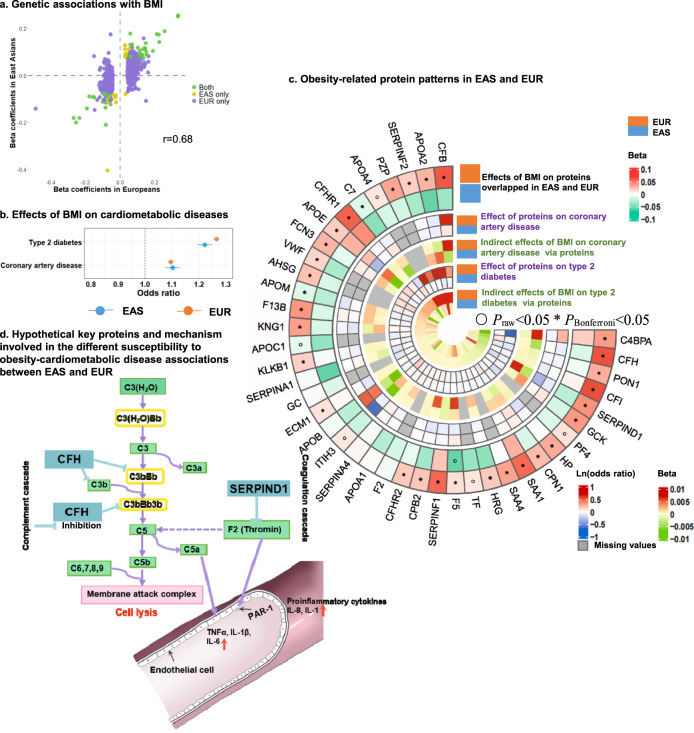
Putative mechanism for difference in BMI-induced type 2 diabetes and coronary artery disease susceptibility between Europeans and East Asians. The analysis comprised 41 proteins with pQTLs in two populations. **a** Shared genetic architecture among two populations. EAS, East Asian; EUR, European. **b** Effect of BMI on cardiometabolic disease risk. The effect sizes are present as odds ratios per 1 kg/m^2^ increase in BMI. The dots represent the estimated effect size, and the lines represent the 95% confidence intervals. **c** Overview of obesity-related protein patterns. The circular heatmap exhibits the effects of proteins on risk of CAD and T2D and indirect effect of the BMI on CAD and T2D via each protein. All statistical tests were two-sided. ^○^*P*_raw_ < 0.05; *P_Bonferroni_ < 0.05. **d** Hypothetical mechanism for susceptibility differences in cardiometabolic diseases between Europeans and East Asians.

With these newly identified pQTL data, we then investigated the potential underlying etiological differences in the susceptibility to cardiometabolic diseases between Europeans and East Asians, given that East Asians compared to Europeans are more susceptible to cardiometabolic diseases at a lower BMI^40–42^. To find a potential interpretation for this phenomenon, we clumped genetic instruments of BMI from the European and East Asian populations, respectively (LD *r*^2^ < 0.05)^43,44^. Correlation analysis indicated a shared genetic architecture between Europeans and East Asians (*r* = 0.68, Fig. 7a). We found that genetically determined BMI was positively associated with T2D and CAD risk across populations, with odds ratio for T2D: 1.22 (95% confidence interval (CI): 1.19–1.25) per 1 kg/m^2^ higher BMI for East Asians, and 1.26 (95% CI: 1.25–1.28) for Europeans. For CAD, the odds ratio was 1.10 (95% CI: 1.08–1.13) for East Asians, and 1.09 (95% CI: 1.09–1.10) for Europeans (Fig. 7b). Thus, in Europeans and East Asians, genetically determined BMI levels had a consistent effect on T2D and CAD.

Using the 41 proteins having pQTLs in both Chinese (our own dataset, representing East Asians) and European populations, we observed that genetically determined BMI was positively associated with 28 proteins and negatively associated with 2 proteins in Europeans (*P* < 0.0006, i.e., 0.05/82 computed by 41 proteins across two populations, Fig. 7c and Supplementary Data 14); in East Asians, however, we found no evidence of above associations after multiple testing corrections, while 34 proteins showing non-significant negative associations. These results suggested that the obesity–protein associations might be substantially different between the two populations with different ancestries.

Next, the effect of the proteins on T2D and CAD was examined. We used publicly available GWAS summary data of T2D and CAD from the European and East Asian populations respectively for our analysis^27,28,45,46^. Using the GSMR method, we found that the negative associations of HP and heparin cofactor 2 (SERPIND1) with CAD and T2D, were consistent between East Asians and Europeans (Fig. 7c and Supplementary Data 14).

To estimate the indirect effect of BMI on T2D and CAD via identified proteins, we performed a two-step MR analysis and used the “product of coefficients” method^47^. We found that HP, SERPIND1, Factor H (CFH), and C4b-binding protein alpha chain (C4BPA) may suppress the effect of BMI on T2D in European populations but not in East Asians (Fig. 7c). Furthermore, in European populations, HP, SERPIND1, CFH, inter-alpha-trypsin inhibitor heavy chain H3 (ITIH3), and kininogen-1 (KNG1) may suppress the effect of BMI on CAD, but not in East Asians. The proteins SERPIND1, KNG1, C4BPA, and CFH were involved in the complement system and blood coagulation, which could regulate the production of proinflammatory cytokines such as tumor necrosis factor (TNF), interleukin-6 (IL-6), interleukin-8 (IL-8) and interleukin-1 (IL-1) (Fig. 7d)^48^. The increased levels of proinflammatory cytokines were associated with higher risk of cardiometabolic diseases, including T2D and cardiovascular diseases^49,50^. Taken together, we discovered a differential obesity-induced proteomics signatures between Europeans and East Asians, which might potentially contribute to the interpretation of different cross-ancestry cardiometabolic disease susceptibilities to obesity status.

## Discussion

MS is a commonly used technique in proteomics research within the biomedical field, with an advantage of not relying on conserved binding regions of the protein target, enabling the discovery of novel protein biomarkers. To the best of our knowledge, however, only a small number of human cohorts have integrated MS-based proteomics with human genetic data^21,51^. Leveraging MS-based methods with a multistage strategy in 2958 Han Chinese participants, we identified 195 lead gene variant–protein associations in total, depicting the genetic architecture of circulating proteins in East Asians. Furthermore, we revealed the potential causal relationships between circulating protein levels and clinically relevant phenotypes in East Asians.

We have also identified novel *trans*-pQTLs that may play a pivotal role in establishing functional links between downstream effectors (i.e., proteins) and disease endpoints, and reveal previously unidentified pathways relevant to disease processes and etiology while their effects on protein levels are mild. However, the physiological significance of the above-mentioned *trans*-pQTLs is yet to be confirmed via in vitro or in vivo perturbation experiments.

From a drug-discovery perspective, the current study provided putative druggable targets for multiple diseases, particularly the novel ones that have not been prioritized in Europeans. Despite proteins are the most common biological class of drug targets, there has been mainly lacking research on non-EUR populations for new drug development. This study, based on East Asians and high-throughput proteomic technologies, enabled a greater understanding of the genetic control of circulating levels of protein drug targets and biomarkers and thereby, may improve pharmaceutical interventions and clinical trials in non-European populations.

The cross-ancestry analyses revealed several proteins that may be crucial for the development or suppression of BMI-induced T2D and CAD, thereby potentially explained differences in the disease susceptibility between Europeans and East Asians. Interestingly, in the population of European ancestry, BMI was mainly positively related to the identified circulating proteins, whereas the negative associations of BMI with these circulating proteins were observed in East Asians. Of these identified proteins, Factor H (CFH) has been reported to regulate the alternative pathway of complement via inactivating C3b and increasing the dissociation of C3 convertase and C5 convertase^52^. A higher level of CFH could reduce inflammation and the formation of immune complex^53^. Another example is heparin cofactor 2 (SERPIND1), a serine proteinase inhibitor that suppresses the functions of thrombin and chymotrypsin^54^. Thrombin exerts proinflammatory effects via promoting complement system activation by cleaving C5 into C5a^55^, or activating protease-activated receptors (PARs), which stimulates the production of IL-8 and IL-1^48^. However, the influence of different proteomics technologies (i.e., SOMAscan and MS) used in the two populations should be considered. Although it will be advantageous to evaluate the impact of ancestry differences by measuring blood proteomics using the same approach in two populations, this sort of dataset was not available at this stage to our knowledge. Thus, the detailed mechanism underlying the relationship among these proteins, obesity, T2D and CAD warrants further investigations.

In summary, the pQTLs identified and analyzed in this study provide an unprecedented resource to unveil the genetic architecture of blood proteome in Han Chinese populations. The newly discovered protein-disease relationships from MR analysis may shed light on the development of novel drug targets for human complex diseases. This is a unique resource for the cross-ancestry evaluation of protein-targeted drug discovery. We also created a web server as an interactive online resource (https://omics.lab.westlake.edu.cn/data/proteins/) for the search and visualization of summary statistics for genetic variants across all measured proteins and peptides.

## Methods

### Ethics approval and consent to participate

The study protocol of Guangzhou Nutrition and Health study was approved by the Ethics Committee of the School of Public Health at Sun Yat-sen University and Ethics Committee of Westlake University. The study protocol of Westlake Precision Birth Cohort was proved by the Ethics Committee of Westlake University. All participants provided written informed consent.

### Study participants and sample collection

The discovery cohort data were derived from the Guangzhou Nutrition and Health study^56,57^. Together, it included up to 2410 participants after excluding related individuals (genetic relatedness >0.05). Included participants were 40–83 years old, living in urban Guangzhou city. Biological samples and questionnaires of the GNHS study were collected at the time of recruitment (2008–2013) and follow-up was scheduled every 3 years. Whole blood samples were collected after overnight fasting. Subsequently, serum and buffy coat separated from whole blood were stored at −80 °C.

### Circulating proteomics profiling

Peptides were extracted from the serum samples as previously described^58^. Briefly, 1 µL of serum samples were lysed using 20 µL of lysis buffer with 8 M urea (Sigma, #U1230) in 100 mM ammonium bicarbonate (ABB) at 32 °C for 30 min. Then the lysates were reduced and alkylated with 10 mM tris (2-carboxyethyl) phosphine (TCEP, Sigma #T4708) and 40 mM iodoacetamide (IAA, Sigma, #SLCD4031). The solution was diluted with 70 µL 100 mM ABB and then a 2-step overnight tryptic digestion (Hualishi Tech. Ltd, Beijing, China), at an enzyme/substrate ratio of 1:60 for 4 h and 12 h, successively. Thereafter, the digestion was quenched with 1% trifluoroacetic (Thermo Fisher Scientific, #T/3258/PB05) to pH 2–3. Peptides were cleaned using C18 SOLAu columns (Thermo, #60209-001) before MS analysis. Peptide samples were then analyzed by SWATH-MS over a 20 min linear LC gradient on a TripleTOF 5600 system (SCIEX, CA, USA) coupled to Eksigent NanoLC 400 System (Eksigent, Dublin, CA, USA). The SWATH-MS method is composed of a 100 ms of full TOF MS scan with an acquisition range of 350–1250 *m/z*, followed by 55 sequential MS/MS scans of variable *m/z* isolation windows from 100 to 1500 Da. The accumulation time was set at 30 ms per isolation window, resulting in a total cycle time of 1.9 s.

After SWATH acquisition, the wiff files were analyzed using DIA-NN (1.7.12)^59^ against a serum spectral library containing 3474 peptide precursors and 536 unique proteins from Swiss-Prot database of Homo Sapiens^60^. In the DIA-NN setting, the peptide length range was set from 5 to 30, the precursor *m/z* range was set from 400 to 1200, and the fragment ion *m/z* range was set from 100 to 1500. The retention time extraction window was automatically set by the software, and the *m/z* extraction window for MS1 and MS2 was 20 ppm and 50 ppm, respectively. Protein and peptide FDRs were controlled below 1%.

### Quality control of proteome analysis

The quality of proteomic data was ensured at multiple steps separately. Proteomic matrix contained missing values. Missing values can be due to the low abundance in certain samples or technical issues. First, to remove the proteomic data with poor quality, we excluded the data with protein identifications below 80% of the median value. Subsequently, we removed the peptide sequences with missingness over 80%. This strategy was aimed to exclude peptide sequences that can only be identified in a small number of samples, which might be false-positive signals due to technical issues. For 1394 biological replicates (i.e., duplicated samples per serum specimen that were randomly selected from all participants), the median Pearson correlation coefficient was 0.973; whereas for 5766 technical replicates (i.e., replicates were acquired with randomly repeated measurements of per prepared sample including unique and duplicated ones), it was 0.965, indicating high reproducibility of proteomics workflow. Then we filled the missing values with each other and calculated mean value for the quantitative results of replicates with a Pearson correlation higher than 0.8 as the final quantitative result of the sample.

### Genotyping data

DNA was extracted from leukocyte using the TIANamp® Blood DNA Kit as per the manufacturer’s instruction. DNA concentrations were determined with the Qubit quantification system (Thermo Scientific, Wilmington, DE, USA). Extracted DNA was stored at −80 °C. Illumina ASA-750K arrays were applied for genotyping. We removed the SNPs with HWE *P* value <0.00001 and missing call rate > 0.05 (Supplementary Data 1). The genetic relationship matrix generated from the LD-pruned (*r*^2^ < 0.2) autosomal SNPs (*n* = 109,079) with GCTA-GREML was used to compute the principal components and cryptic relatedness. Individuals with a high or low proportion of heterozygous genotypes (outliers defined as 3 standard deviations), sex mismatch, or different ancestries (the first two principal components ±5 standard deviation from the mean) were excluded^61^. After that, genetic variants were mapped to the 1000 Genomes Project Phase3 v5 by SHAPEIT^62,63^, and then imputed with 1000 Genomes Project Phase3 v5 reference panel by Minimac3^64,65^. We included genetic variants with imputation accuracy RSQR >0.3 and MAF >0.05 for the GWAS analyses.

### Genome-wide association analysis in the discovery cohort (Guangzhou Nutrition and Health Study)

In the main model, we replaced the missing data by 1/2 of the minimum observed value in the protein or peptide matrix. The abundances of proteins were rank inverse normalized, and then we applied the GWAS analysis at four measurement batches according to the time of finishing the measurements of the proteome (here we called them four sub-cohorts). In each measurement batch (sub-cohort), a mixed linear model (MLM)-based association analysis was performed with GCTA-MLMA^16,17^, adjusted for the covariates including age, sex, and the first five genetic principal components of ancestry as fixed effects and the effects of all the SNPs as random effects.

### Meta-analysis of genome-wide association studies

GWAMA software was used to perform a meta-analysis of our serum proteome GWAS analyses across the four sub-cohorts based on a random-effect model^66^. The genome-wide significant associations that should have (i) meta-analysis *P* < 5 × 10^−8^/*n*, where *n* is the number of proteins or peptide precursors used for the analysis; (ii) *P* < 0.05 in four sub-cohorts; (iii) consistent direction of effect across the sub-cohorts.

### Power calculation

The genetic association has a test statistic which is a chi-square distribution with one degree of freedom. It is a non-central chi-square distribution under the alternative hypothesis, while it is a central chi-square distribution under the null alternative. We calculated the non-centrality parameter (NCP) by ${{{{{\rm{NCP}}}}}}=\frac{2f\left(1-f\right){b}^{2}N}{1-2f\left(1-f\right){b}^{2}}$, where *N* is the sample size, *f* is the allele frequency and *b* is the estimated value of the GWAS analysis. The test statistic of a central chi-square distribution with one degree of freedom is ${{{{{\rm{t}}}}}}={F}^{-1}(1-p,1)$, where *F* is the cumulative distribution function of a central chi-square distribution with one degree of freedom and *p* is the significance threshold of GWAS analysis. The statistical power is ${{{{{\rm{P}}}}}}=1-{{{{{\rm{G}}}}}}({{{{{\rm{t}}}}}},{{{{{\rm{NCP}}}}}},1)$, where G is the cumulative distribution function of a non-central chi-square distribution with one degree of freedom.

### Conditional analysis

To identify secondary signals at the identified loci, conditional analysis was implemented with GCTA-COJO^18^ at a stepwise selection procedure for both identified proteins and peptides with the threshold of *P* < 1.6 × 10^−10^ or 3.9 × 10^−11^, respectively. Linkage disequilibrium (LD) was estimated in 2536 unrelated participants from the discovery study.

### Heritability analysis

The SNP heritability was estimated according to the procedures described by the previous study^5^. First, phenotypic variance explained by the lead pQTLs was calculated by 2β^2^MAF(1−MAF), where β was the effect size of the genetic variance and MAF represented the minor allele frequency. Given that the LDSC regression performed poorly when large effect genes were present and the variance explained by the major loci could be double-counted via LD, the contribution of the polygenic background was estimated in SNPs other than the genetic variants located within 10 Mb of the lead pQTLs. We used the LDSC regression to estimate the contribution of the polygenic background for the proteins with genome-wide significant associations^67^.

In addition, to capture the variance explained by the SNPs jointly associated with the lead SNPs, we used the formula q_j_^2^ = 2 × β × β_j_ × MAF × (1 − MAF), where β_j_ was the estimate from the conditional analysis.

### Functional annotation

A pQTL was defined as *cis* when it was located within 1 Mb distance of the transcript starting site (TSS). TSSs of proteins were accessed from Ensembl GRCh37 Version 102 by the UniProtKB ID (using “biomaRt” R package). For all identified loci, the predicted function was annotated with Ensembl VEP release 104^68^. The nearest gene of each locus was annotated with GENCODE Version 29, using BEDOPS (“closest-features” function)^69,70^. Expression profiles for proteins in tissues were based on The Human Protein Atlas Version 20.1 and Ensembl version 92.38^33,34^.

### Replication analysis of the previously identified pQTLs in the literature

To compare our results with those in the previous studies using different techniques^5,8,12,19–21^, based on the discovery cohort, we tried to replicate those previously identified associations. After removing the complementary bases in consideration of flipping strands, genetic associations were considered to be replicated up to the criteria: (i) significant *P* value after FDR correction; (ii) consistent direction of effect.

### Replication analysis of the novel identified pQTLs

To test whether the novel identified pQTLs in our discovery cohort could be replicated in an independent cohort, we assessed them in the Westlake Precision Birth Cohort (WEBIRTH), consisting of 548 gestational women aged 21–44 years old (ClinicalTrials.gov Identifier: NCT04060056). Serum proteome profiling and processing of genetic data were performed with the identical pipeline as the discovery cohort. We excluded one of each pair of participants with estimated genetic relatedness >0.05. After that, the protein abundances were rank inverse normalized and performed with the GWAS analysis using GCTA-MLMA, adjusted for covariates including age, gestational week, and the first five genetic principal components of ancestry.

### Colocalization of pQTLs with clinically relevant phenotypes

To investigate the genetic correlation of the circulating protein levels with the clinically relevant phenotypes, we performed the multi-SNP-based SMR (summary-data-based Mendelian randomization) test and HEIDI (heterogeneity in dependent instruments) analysis in the Asian populations^23,24,30^, using the *cis*-pQTLs as the exposure variables and drawing the outcome SNPs from the Biobank Japan (BBJ) study as well as GWAS summary statistics of type 2 diabetes from the AGEN-T2D study^25–28^. The reference sample was 2536 unrelated participants from the GNHS study. We excluded the SNPs located in the MHC region (chr6:28,477,797–33,448,354) due to the complexity of this region and included the gene probes or proteins with at least *cis*-acting variants at *P* < 5 × 10^−8^. The significance at Bonferroni correction <0.05 and acceptance by the HEIDI test (*P*_HEIDI_ > 0.05) were both required to be recognized as significant.

### Mendelian randomization analysis

We performed a bi-directional two-sample Mendelian randomization (MR) analysis with *cis*-pQTLs (LD *r*^2^ < 0.05) clumped at *P* < 5 × 10^−8^ and all identified *trans*-acting variants. The outcome variables were obtained from the aforementioned studies including BBJ and the AGEN-T2D study^25–28^. We reported the associations passing the Bonferroni correction at *P-*corrected <0.05. GSMR (Generalized Summary-data-based Mendelian Randomization)^30^ was used for the bi-directional MR analysis. For each trait included in the reverse MR analysis, the independent instrumental variables (LD *r*^2^ < 0.05) were clumped at *P* < 5 × 10^−8^ in PLINK^71^.

### Mediation analysis

We used a two-step MR approach with GSMR to investigate the effect of BMI on T2D via proteins. First, we evaluated the total effect of BMI on T2D based on summary-level GWAS results^28,43,44,46^. Given that the BMI s.d. was larger in the European populations (s.d. = 3.7 in the Biobank Japan study, 4.65 in the GIANT study), we converted 1-SD unit to 1 kg/m^2^ unit. Then the MR analysis for the effect of BMI on proteins (${{{{{\rm{\alpha }}}}}}$) and the effect of proteins on T2D (${{{{{\rm{\beta }}}}}}$) to estimate the indirect effects with “product of coefficients”^47^. The standard errors for the indirect effects were derived as the formula ${\sigma }_{\alpha \beta }=\sqrt{{\alpha }^{2}{\sigma }_{\beta }^{2}+{\beta }^{2}{\sigma }_{\alpha }^{2}-{\sigma }_{\alpha }^{2}{\sigma }_{\beta }^{2}}$.

### Reporting summary

Further information on research design is available in the Nature Portfolio Reporting Summary linked to this article.

## Supplementary information

Description of Additional Supplementary Files

Supplementary Data 1

Supplementary Data 2

Supplementary Data 3

Supplementary Data 4

Supplementary Data 5

Supplementary Data 6

Supplementary Data 7

Supplementary Data 8

Supplementary Data 9

Supplementary Data 10

Supplementary Data 11

Supplementary Data 12

Supplementary Data 13

Supplementary Data 14

Supplementary Data 15

Reporting Summary

## Data Availability

An interactive web resource (https://omics.lab.westlake.edu.cn/data/proteins) was developed to visualize our pQTL data. The raw data for serum proteomics are available in the iProX (https://www.iprox.cn/page/home.html) at accession numbers PXD039236, PXD039231, and PXD038253. Other datasets generated during and/or analyzed during this study are available upon reasonable request by bona fide researchers for specified scientific purposes via contacting the corresponding authors.

## Code availability

Analysis code is available via: https://github.com/nutrition-westlake/Chinese_pQTL/blob/main/Data_analysis.
