# Supplementary material for: Genome-wide genotype-serum proteome mapping provides insights into the cross-ancestry differences in cardiometabolic disease susceptibility
Source: Nat Commun. 2023 Feb 16;14:896. doi: 10.1038/s41467-023-36491-3 (PMC9935862; doi:10.1038/s41467-023-36491-3)
Supplement: Supplementary file 1 — Description of Additional Supplementary Files [file 41467_2023_36491_MOESM1_ESM.pdf]

Description of Additional Supplementary Files

File Name: Supplementary Data 1
Description: Characteristics of included studies
File Name: Supplementary Data 2
Description: Information of proteins and peptides measured by DIA-MS
File Name: Supplementary Data 3
Description: Summary for identified pQTLs
File Name: Supplementary Data 4
Description: Summary for the heritability analysis
File Name: Supplementary Data 5
Description: Summary for the conditional analysis
File Name: Supplementary Data 6
Description: Replication of identified loci in the independent external studies
File Name: Supplementary Data 7
Description: Replication of the previously reported pQTLs
File Name: Supplementary Data 8
Description: Colocalization of cis-pQTLs and clinically relevant phenotypes
File Name: Supplementary Data 9
Description: Mendelian randomization analysis
File Name: Supplementary Data 10
Description: Genetic instruments for Mendelian randomization analysis
File Name: Supplementary Data 11
Description: Reverse Mendelian randomization
File Name: Supplementary Data 12
Description: Identified gene-protein-phenotype associations
File Name: Supplementary Data 13
Description: Drug target information
File Name: Supplementary Data 14
Description: Cross-ancestry analysis for cardiometabolic disease susceptibility
File Name: Supplementary Data 15
Description: Important protein-phenotype associations identified by colocalization and Mendelian randomization analyses
