# Supplementary material for: Genome-wide genotype-serum proteome mapping provides insights into the cross-ancestry differences in cardiometabolic disease susceptibility
Source: Nat Commun. 2023 Feb 16;14:896. doi: 10.1038/s41467-023-36491-3 (PMC9935862; doi:10.1038/s41467-023-36491-3)
Supplement: Supplementary file 17 — Reporting Summary [file 41467_2023_36491_MOESM17_ESM.pdf]

nature portfolio

Corresponding author(s): Ju-Sheng Zheng
Last updated by author(s): Jan 11, 2023

Reporting Summary

Nature Portfolio wishes to improve the reproducibility of the work that we publish. This form provides structure for consistency and transparency in reporting. For further information on Nature Portfolio policies, see our Editorial Policies and the Editorial Policy Checklist.

Statistics

For all statistical analyses, confirm that the following items are present in the figure legend, table legend, main text, or Methods section.

n/a	Confirmed	
☐	☒	The exact sample size (*n*) for each experimental group/condition, given as a discrete number and unit of measurement
☐	☒	A statement on whether measurements were taken from distinct samples or whether the same sample was measured repeatedly
☐	☒	The statistical test(s) used AND whether they are one- or two-sided<br>*Only common tests should be described solely by name; describe more complex techniques in the Methods section.*
☐	☒	A description of all covariates tested
☐	☒	A description of any assumptions or corrections, such as tests of normality and adjustment for multiple comparisons
☐	☒	A full description of the statistical parameters including central tendency (e.g. means) or other basic estimates (e.g. regression coefficient) AND variation (e.g. standard deviation) or associated estimates of uncertainty (e.g. confidence intervals)
☐	☒	For null hypothesis testing, the test statistic (e.g. *F*, *t*, *r*) with confidence intervals, effect sizes, degrees of freedom and *P* value noted<br>*Give P values as exact values whenever suitable.*
☒	☐	For Bayesian analysis, information on the choice of priors and Markov chain Monte Carlo settings
☐	☒	For hierarchical and complex designs, identification of the appropriate level for tests and full reporting of outcomes
☐	☒	Estimates of effect sizes (e.g. Cohen's *d*, Pearson's *r*), indicating how they were calculated

Our web collection on statistics for biologists contains articles on many of the points above.

Software and code

Policy information about availability of computer code

Data collection	DIA-NN (v.1.7.12) was used to analyze the proteomics data.
Data analysis	GCTA (v.1.92.4)<br>SHAPEIT (v.2.r904)<br>Minimac3 (v.2.0.1)<br>LDSC (v.1.0.1)<br>BEDOPS (v.2.4.38)<br>smr_Linux (v.1.03)

For manuscripts utilizing custom algorithms or software that are central to the research but not yet described in published literature, software must be made available to editors and reviewers. We strongly encourage code deposition in a community repository (e.g. GitHub). See the Nature Portfolio guidelines for submitting code & software for further information.

Data

Policy information about availability of data

All manuscripts must include a data availability statement. This statement should provide the following information, where applicable:

- Accession codes, unique identifiers, or web links for publicly available datasets
- A description of any restrictions on data availability
- For clinical datasets or third party data, please ensure that the statement adheres to our policy

Full GWAS summary statistics for pQTLs are available at https://omics.lab.westlake.edu.cn/data/proteins. Analysis code is available via: https://github.com/nutrition-westlake/Chinese_pQTL/blob/main/Data_analysis. The mass spectrometry proteomics data have been deposited to the ProteomeXchange Consortium (http://proteomecentral.proteomexchange.org). The proteomics data are available upon reasonable request by bona fide researchers for specified scientific purposes via contacting the corresponding authors.

Human research participants

Policy information about studies involving human research participants and Sex and Gender in Research.

Reporting on sex and gender	We determined the sex information based on the genotyping array data.
Population characteristics	The Guangzhou Nutrition and Health study is a community-based prospective cohort study in Guangzhou city, China (mean age 63.4 (6.4), 30% male). Participants in the Westlake Precision Birth Cohort are gestational women (mean age 31.1(3.8)). We reported more details in Supplementary Table 1.
Recruitment	The Guangzhou Nutrition and Health study is a community-based prospective cohort study in Guangzhou city, China. The Westlake Precision Birth Cohort recruited gestational women aged 21 to 44 years old.
Ethics oversight	The study protocol of Guangzhou Nutrition and Health study was approved by the Ethics Committee of the School of Public Health at Sun Yat-sen University and Ethics Committee of Westlake University. The study protocol of Westlake Precision Birth Cohort was proved by the Ethics Committee of Westlake University. All participants provided written informed consent.

Note that full information on the approval of the study protocol must also be provided in the manuscript.

Field-specific reporting

Please select the one below that is the best fit for your research. If you are not sure, read the appropriate sections before making your selection.

- [x] Life sciences
- [ ] Behavioural & social sciences
- [ ] Ecological, evolutionary & environmental sciences

For a reference copy of the document with all sections, see nature.com/documents/nr-reporting-summary-flat.pdf

Life sciences study design

All studies must disclose on these points even when the disclosure is negative.

Sample size	After applying data exclusion criteria, a total sample size of 2,410 individuals was used. The sample size was chosen based on the data that were available when the study was launched since no prior sample size estimates had been made.
Data exclusions	The genetics exclusion criteria for the study is the following: (1) high or low proportion of heterozygous genotypes (outliers defined as 3 standard deviations); (2) sex mismatch; (3) different ancestries (the first two principal components ±5 standard deviation from the mean).
Replication	We replicated the identified signals in a independent cohort study (Westlake Precision Birth Cohort).
Randomization	There was no grouping of participants in the study because it was a population-based study.
Blinding	There was no blinding during the sample collection.

Reporting for specific materials, systems and methods

We require information from authors about some types of materials, experimental systems and methods used in many studies. Here, indicate whether each material, system or method listed is relevant to your study. If you are not sure if a list item applies to your research, read the appropriate section before selecting a response.

Materials & experimental systems

n/a	Involved in the study
☒	☐ Antibodies
☒	☐ Eukaryotic cell lines
☒	☐ Palaeontology and archaeology
☒	☐ Animals and other organisms
☒	☐ Clinical data
☒	☐ Dual use research of concern

Methods

n/a	Involved in the study
☒	☐ ChIP-seq
☒	☐ Flow cytometry
☒	☐ MRI-based neuroimaging
